# Together we can counter the effects of climate change

**DOI:** 10.25646/11399

**Published:** 2023-06-01

**Authors:** Gerhard Adrian, Martin Dietrich, Birgit Esser, Andreas Hensel, Folkhard Isermeyer, Dirk Messner, Thomas C. Mettenleiter, Inge Paulini, Sabine Riewenherm, Lars Schaade, Ralph Tiesler, Lothar H. Wieler

**Affiliations:** 1 German Meteorological Service; 2 Federal Centre for Health Education; 3 German Federal Institute of Hydrology; 4 German Federal Institute for Risk Assessment; 5 Thünen Institute; 6 German Environment Agency; 7 Friedrich-Loeffler-Institut; 8 Federal Office for Radiation Protection; 9 Federal Agency for Nature Conservation; 10 Robert Koch Institute; 11 Federal Office of Civil Protection and Disaster Assistance; 12 Formerly Robert Koch Institute

Climate change is the greatest challenge facing humanity, threatening our livelihoods and our secure future. The impact of anthropogenic environmental change on human health and well-being is increasing. Public health systems worldwide need to address this significant and complex burden by strengthening both their capacity to act and their resilience.

As highlighted in the Roadmap of the International Association of National Public Health Institutes (IANPHI) and supported by the G7 health ministers in a communiqué, national public health institutes have a key role to play in climate change mitigation and adaptation [1, 2]. Nutrition and mobility are particularly relevant in this context, as health-promoting behaviour in these fields simultaneously aids climate protection, as does the transformation to sustainable and resilient (public) health systems. Within the framework of the German Strategy for Adaptation to Climate Change (DAS), health is an important topic when considering effective and sustainable measures for dealing with the climate crisis. Climate change affects many other fields that intersect with health, such as water management, construction or urban and regional development. Therefore, health-sensitive climate protection and climate adaptation require intersectoral cooperation and the continuous exchange between different actors in line with the ideas behind One Health and Health in All Policies [3, 4].

In this context, the German Status Report on Climate Change and Health is an important project that can help to address the health challenges of the climate crisis and to strengthen the cooperation between different institutions and authorities. We, the leaders of public authorities in Germany working on public health issues, consider interdisciplinary and intersectoral cooperation to be a key prerequisite for best addressing the health challenges of climate change. This implies the need for innovative and cooperative collaboration between different sectors, not only at the municipal, state and federal level, but also in terms of exchange between these levels.

The German Status Report on Climate Change and Health 2023 is published in an article series in the Journal of Health Monitoring in three issues.

The first issue begins with an introductory article outlining the range of topics covered in the status report, and devotes four thematic articles to the influence of climate change on infectious diseases (vector- and rodent-borne diseases, waterborne infections and intoxications, foodborne infections and intoxications) and antimicrobial resistance.

In the second issue, six articles describe the influence of climate change on non-communicable diseases caused by heat and other extreme weather events such as floods, by increased UV radiation, by allergic diseases and by increased air pollution. The impact of climate change on mental health is also discussed.

The findings from these first two issues are incorporated into the contributions of the final issue. They examine health equity with regard to the effects of climate change, highlight the importance of target group-specific climate change communication, and make concluding recommendations for action based on those formulated in the other articles.

In addition to the various topic-specific recommendations, all contributions have one thing in common: they point to a continuing need for research. Extended monitoring of many of the health effects of climate change is also recommended. The cooperation of the authorities and research institutions dedicated to these important tasks is crucial.

We hope that this report will be an important step towards even better cooperation between science and decision-makers in politics and society.

## References

[1] International Association of National Public Health Institutes (2021) IANPHI roadmap for action on health and climate change: Engaging and supporting national public health institutes as key climate actors. https://ianphi.org/news/2021/roadmap-climate-change.html (As at 30.03.2023)

[2] G7 (2022) Communiqué of the G7 Health Ministers, 20 May 2022, Berlin. https://www.bundesgesundheitsministerium.de/fileadmin/Dateien/3_Downloads/G/G7/20220520_German_G7_Health_Ministers_Communique.pdf (As at 30.03.2023)

[3] One Health High-Level Expert Panel (2022) One Health: A new definition for a sustainable and healthy future. PLoS Pathog 18(6):e1010537 35737670 10.1371/journal.ppat.1010537 PMC9223325

[4] World Health Organization (2023) Promoting Health in All Policies and intersectoral action capacities. https://www.who.int/activities/promoting-health-in-all-policies-and-intersectoral-action-capacities (As at 30.03.2023)

